# Supplementary material for: Exploring health anxiety and its association with quality of life in Yemeni medical undergraduates: A national cross-sectional
Source: PLoS One. 2026 Apr 6;21(4):e0346296. doi: 10.1371/journal.pone.0346296 (PMC13052843; doi:10.1371/journal.pone.0346296)
Supplement: Attachment — Submitted filename: Review coments - PONE.docx [file pone.0346296.s002.docx]

**REVIEW COMMENTS**

This paper tackles a relevant issue; however weak study methods could cast doubt on what was observed. How data were collected is particularly important as it influences how reliably conclusions hold up across different groups.

**Methodology**

Methodological Strengths

The use of trusted tools like the Arabic SF-12 and SHAI questionnaires which have been validated.

Strong psychometric properties have been reported for both measures.

Efforts were spread across data collection platforms to lower the chance of biased outcomes.

People joined without being known, this helped to keep answers honest because nobody wanted to impress others.

**Critical Issues**

1.A closer look at the methodology section reveals some problems with how the data samples were chosen. A small group was chosen without random selection, meaning findings might not apply more widely.

This might make students who engage more strongly conspicuous.

Sampling by ease could fall short of capturing full diversity: geographic limits matter even when ten medical schools joined.

Study Design Section

Design Strengths:

The use of the multi-institutional approach (ten medical schools) enhances scope of the study.

Reliability is boosted by using uniform methods to gather information.

People made the decision to take participate voluntarily so the effort stays honest and fair.

Looking back critically, key issues stand out.

Critical Design Flaws:

1. Sampling Design Issues:

Convenience sampling fundamentally limits the study's external validity.

Geographic dispersion challenges not adequately addressed in the original design.

2. Data Collection Design:

The choice to go with self-reported surveys might have tilted the responses to one direction.

Looking at many things all at once might influence knowing cause from effect.

3. Analytical Framework:

Design Strengths:

Multi-institutional approach (ten medical schools) enhances scope

Using uniform methods to gather information boosts reliability

Students had the choice to take part so the effort stays honest and fair

Use of validated instruments which are psychometrically sound, culturally adapted tools

The authors acknowledged that their method was not perfect and took steps to lower biases where feasible.

The Large group of authors suggests a collaborative multi-institutional approach.

Looking back critically, key issues stand out:

Critical Issues

Tables without clear details point to flaws in how they are being displayed

The results might not apply widely to medical school students across Yemen because participants were recruited easily. This weakens the conclusions that can be drawn.

Two tools were used and this might not be enough when looking at how health worry and daily life connect. A single approach could miss key patterns or effects. Though useful, relying on only two key measures limits what can be learnt about this complex link.

Taking a fresh look at data could work better if different groups are included, maybe blending quantitative and qualitative might give a fuller picture of the findings.

The use of varied assessment methods can enhance analysis depth and deepen understanding.

Methods section should be outlined clearer to include specific steps taken during research activities

Include sample size justification and power calculations to support statistical power analysis.
