# Supplementary material for: Exploring health anxiety and its association with quality of life in Yemeni medical undergraduates: A national cross-sectional
Source: PLoS One. 2026 Apr 6;21(4):e0346296. doi: 10.1371/journal.pone.0346296 (PMC13052843; doi:10.1371/journal.pone.0346296)
Supplement: Attachment — Submitted filename: Point to point response to reviewer comments.docx [file pone.0346296.s001.docx]

**Point to point response**

**Reviewer #1:**

***Comment 1:***
Literary editing is recommended to use some common scientific terms.

***Response 1:***

Thank you for the valuable feedback. We carefully revised the manuscript to ensure consistent use of standard scientific terminology and improved overall clarity and precision in language.

***Comment 2:***
Have the tools been tested for validity and reliability?

***Response 2:***

Thank you for your comment. The tools used in our study were previously validated and tested for reliability by the original authors. We obtained permission to use these instruments and followed the same standardized procedures as described in the original validation studies. ***Comment 3:***
Have you taken any specific steps to mitigate the limitations of the study?

***Response 3:***

Thank you for your comment. We have addressed the limitations by acknowledging those that could not be mitigated and by taking several steps to minimize potential biases where possible. For example, we used previously validated tools to enhance measurement accuracy, maintained participant anonymity to reduce social desirability bias, and ensured that data collection procedures were standardized to improve consistency. Nonetheless, we transparently reported the remaining limitations that were beyond our control.

***Comment 4:***
The results of some studies presented in the introduction in the discussion section can be helpful.
***Response 4:***

Thank you for your constructive suggestion. We agree that integrating the results of the studies mentioned in the introduction into the discussion section would enhance the depth and coherence of our interpretation. We revised the discussion to include relevant comparisons and contrasts with these studies to better contextualize our findings within the existing literature.

**Reviewer #2:**

***Comment 1:***
Despite the interesting topic undertaken by such a large group of co-authors, she wanted to highlight shortcomings such as too few research tools, too limited discussion, and incompletely described tables.

***Response 1:***
Thank you for your valuable feedback. We appreciate your positive note on the study topic and the collaborative effort. We acknowledge the mentioned shortcomings and will address them accordingly. Specifically, we planned to clarify and expand the description of the research tools, enrich the discussion section to provide deeper analysis and stronger connections with existing literature, and ensure that all tables are fully described and clearly presented for better understanding.

**Reviewer #3:**

Introduction
***Comment 1:***
The introduction should address the quality of life of medical students.

***Response 1:***
Thank you for your comment. We appreciate the suggestion. We have already addressed the quality of life of medical students in the introduction, emphasizing its relevance to the study’s objectives and its relationship with health anxiety. ***Comment 2:***
The prevalence of anxiety among medical students in Yemen should be reported.

***Response 2:***
Thank you for your comment. We appreciate the suggestion. In response, we have added the available data on the prevalence of anxiety among Yemeni medical students, noting that anxiety was found to have a prevalence of 34.8% for cases categorized as moderate and severe. This addition strengthens the background and contextual relevance of our study.
 ***Comment 3:***
The manuscript should be reviewed for academic writing structure and grammatical accuracy.
***Response 3:***
Thank you for your comment. We appreciate the feedback. The manuscript has been carefully reviewed and revised to improve academic writing style, structure, and grammatical accuracy, ensuring clarity and consistency throughout the text.

Methods
***Comment 1:*** Provide a more detailed explanation of the sampling method.

***Response 1:*** Thank you for your comment. We have expanded the description of the sampling method to provide more detail. A non-probability convenience sampling approach was used to recruit participants from ten medical schools across Yemen due to feasibility constraints and the geographic dispersion of students. Efforts were made to include a diverse sample in terms of gender, academic year, and location. To reduce selection bias, the survey was distributed through multiple official student channels and widely used social media platforms. Participation was voluntary and anonymous, and informed consent was implied upon submission. We also acknowledge the inherent limitations of this approach, such as potential overrepresentation of more engaged students, which have been discussed in the manuscript.

***Comment 2:*** Describe the ethical considerations.

***Response 2:*** Thank you for your comment. We would like to clarify that the details regarding ethical approval and informed consent are already provided in the “Ethical Approval and Consent to Participate” section of the manuscript, located in the last section for declaration.

***Comment 3:*** Report the validity and reliability of the questionnaire.

***Response 3:*** Thank you for your comment. We would like to clarify that the questionnaires used in this study have established validity and reliability, including their Arabic versions. Specifically:

Arabic SF-12: Internal consistency ranged from α = 0.70–0.84 for the individual components and α = 0.80 for the total score. Test-retest reliability was excellent (ICC = 0.80–0.99), and construct validity was confirmed through a two-factor structure via principal component analysis. It is validated and ready for use.

Arabic SHAI: Internal consistency ranged from α = 0.77–0.85 for the components and α = 0.85 for the total score. Construct validity was supported with a two-factor structure confirmed and found to be gender-invariant. The tool is validated and psychometrically promising.

These instruments were employed in our study as part of a structured, self-administered questionnaire, ensuring reliable and valid measurement of health anxiety and quality of life among participants.

***Comment 4:*** Explain the data collection procedure in detail.

***Response 4:*** Thank you for your comment. We would like to clarify that we have already provided a detailed description of the data collection procedure in the manuscript, as Reviewer 1 asked.

Discussion
***Comment 1:*** In this section, make greater use of participants’ personal history of psychiatric disorders and their personality traits to interpret and explain the results.

***Response 1:*** Thank you for your comment. We would like to clarify that we have already incorporated participants’ personal and family history of psychiatric disorders, as well as relevant personality traits, into the interpretation and discussion of our results to provide a more comprehensive understanding of the findings.

**Reviewer #4:**

The authors present a well-written and interesting manuscript exploring risk and protective factors of health anxiety and its relationship with QoL of medical students in Yemen. The authors have conducted rigorous analysis leading to novel findings. I found the manuscript to be highly relevant for the development of targeted interventions to support medical students.

The study complies with ethical standards, is methodologically sound and clearly reported, and limitations are acknowledged without undermining the study’s validity. I suggest these corrections before publication:

1. For future submissions, please use line numbering and page numbering in the draft document to help smooth the review process.

***Response:*** Thank you for your comment. We acknowledge this recommendation and will ensure that line numbering and page numbering are included in all future draft submissions to facilitate a smoother review process.

2. Methods, study design first line: change “The research” for “This study” or “The current study”.

***Response:*** Thank you for your comment. We have revised the first line of the Methods, Study Design section to replace “The research” with “This study” for improved clarity and consistency.

3. In the Statistical Analysis section, please include a clear description specifying how each outcome variable was analysed.

***Response:*** Thank you for your comment. We have revised the Statistical Analysis section to clearly specify how each outcome variable was analyzed.

4. How were the open-ended questions analyzed and included in the study? It is not clear.
***Response***: Thank you for your comment. We would like to clarify that the questionnaire did not include any open-ended questions; all data were collected using structured, closed-ended items.

5. Health anxiety appears throughout the document as: “Health Anxiety”, “Health anxiety”, “health anxiety| or “HA”, please choose one and review the document for consistency.

***Response***: Thank you for your comment. We have reviewed the manuscript and standardized the terminology for consistency. Throughout the document, “health anxiety” (all lowercase, without quotation marks) is now used uniformly, and the abbreviation “HA” has been removed to avoid confusion.

6. In Table 1, last row, please review the history of psychiatric Disorders so that results are presented consistently with the rest of the table. For example, Family History of Psychiatric Disorders, where data of both “No, I don’t” & “Yes, I do” are presented.

***Response***: Thank you for your comment. We have revised Table 1 to present the history of psychiatric disorders consistently with the other variables, showing both “No” and “Yes” categories with their corresponding data.

7. Please review the words that denote causation or direction throughout the document and replace these with words that denote a relationship or association; for example, “influenced” by “ was related to” or “was linked to”

***Response***: Thank you for your comment. We have reviewed the manuscript and replaced all terms that imply causation with language indicating association or relationship. For example, words such as “influenced,” “caused,” or “led to” have been replaced with phrases like “was related to,” “was associated with,” or “showed an association with” to accurately reflect the cross-sectional nature of the study.

8. Please clarify in the discussion the relationship between health anxiety and academic underachievement. If I understand correctly, both directions are considered – this is, underachievement can contribute to health anxiety, and the other way around – with which I agree, but this needs to be clarified in the text and extended.

***Response***: Thank you for your comment. We would like to clarify that we have already addressed this point in the discussion, highlighting the bidirectional relationship between health anxiety and academic underachievement.

9. In general, the discussion needs to be strengthened.

***Response***: Thank you for your comment. We acknowledge this suggestion and have strengthened the discussion by providing more in-depth interpretation of the findings, integrating relevant literature, and highlighting the implications of health anxiety and quality of life among Yemeni medical students.
