# Supplementary material for: Exploring health anxiety and its association with quality of life in Yemeni medical undergraduates: A national cross-sectional
Source: PLoS One. 2026 Apr 6;21(4):e0346296. doi: 10.1371/journal.pone.0346296 (PMC13052843; doi:10.1371/journal.pone.0346296)
Supplement: Attachment — Submitted filename: Point to point response to reviewer comments 2.docx [file pone.0346296.s003.docx]

**Response to Reviewers**

**Comment 1:** This paper tackles a relevant issue; however weak study methods could cast doubt on what was observed. How data were collected is particularly important as it influences how reliably conclusions hold up across different groups.

**Response:**
We thank the reviewer for highlighting this important concern. In response, we have substantially revised the Methods section to improve clarity, transparency, and rigor. Specifically, we expanded the descriptions of the study design, sampling strategy, data collection procedures, and statistical analysis, and we strengthened the Limitations section to explicitly acknowledge the implications of the chosen methodology on generalizability and causal inference. These revisions ensure that conclusions are appropriately framed and interpreted within the methodological constraints of the study.

**Critical issues**

**1. A closer look at the methodology section reveals some problems with how the data samples were chosen. A small group was chosen without random selection, meaning findings might not apply more widely.
This might make students who engage more strongly conspicuous.
Sampling by ease could fall short of capturing full diversity: geographic limits matter even when ten medical schools joined.**

**Response:**
We acknowledge this limitation and have addressed it directly. The Sampling Strategy subsection has been rewritten to clearly justify the use of non-probability convenience sampling, which was necessitated by practical constraints such as the absence of centralized student registries, wide geographic dispersion, limited resources, and security challenges. We also clarified that recruiting a large sample (n = 2,573) from ten geographically diverse medical schools was intended to enhance heterogeneity. Additionally, the Limitations section now explicitly states that convenience sampling may limit external validity and that findings should be interpreted cautiously with respect to generalizability.

**Critical design flaws**

**1. Sampling design issues:
Convenience sampling fundamentally limits the study's external validity.
Geographic dispersion challenges not adequately addressed in the original design.**

**Response:**
We agree with this assessment. The revised manuscript now explicitly discusses the limitations of convenience sampling in both the Methods and Limitations sections. Geographic dispersion and feasibility challenges are now clearly described, and we explain how multi-institutional recruitment and broad online dissemination were used to partially mitigate these issues, while acknowledging that full representativeness cannot be ensured.

**2. Data collection design:
The choice to go with self-reported surveys might have tilted the responses to one direction.
Looking at many things all at once might influence knowing cause from effect.**

**Response:**
These concerns have been fully addressed. We clarified that self-reported data may be subject to reporting bias and that anonymity and validated instruments were used to minimize this risk. We also explicitly stated in the Study Design and Limitations sections that the cross-sectional nature of the study precludes causal inference, and that observed relationships should be interpreted as associations rather than cause-and-effect relationships.

**Analytical framework**

**Critical issues**

**1. Tables without clear details point to flaws in how they are being displayed**

**Response:**
We have thoroughly revised Tables 1–3 to improve clarity and consistency. Table titles, variable labels, reference categories, statistical tests, and footnotes have been standardized. All tables are now self-contained, clearly presented, and aligned with journal reporting standards.

**2. The results might not apply widely to medical school students across Yemen because participants were recruited easily. This weakens the conclusions that can be drawn.**

**Response:**
We agree and have addressed this directly by tempering conclusions throughout the manuscript. The Abstract, Discussion, and Conclusion sections now explicitly use cautious language and avoid overgeneralization. The Limitations section clearly states that findings may not be fully generalizable to all Yemeni medical students.

3. **Two tools were used and this might not be enough when looking at how health worry and daily life connect. A single approach could miss key patterns or effects. Though useful, relying on only two key measures limits what can be learnt about this complex link.**

**Response:**
This limitation is now explicitly acknowledged in the Limitations section. We note that although the SHAI and SF-12 are validated and appropriate for the study objectives, relying on two quantitative instruments may not capture the full complexity of health anxiety and quality of life. We also suggest that future studies incorporate additional psychosocial measures or mixed-methods approaches.

4. **Taking a fresh look at data could work better if different groups are included, maybe blending quantitative and qualitative might give a fuller picture of the findings.
The use of varied assessment methods can enhance analysis depth and deepen understanding.**

**Response:**
We fully agree and have incorporated this recommendation into the Limitations and Future Directions discussion, emphasizing the value of longitudinal and mixed-methods research to enhance depth and causal understanding.

**5. Methods section should be outlined clearer to include specific steps taken during research activities**

**Response:**
The Methods section has been comprehensively rewritten and reorganized with clear subheadings, step-by-step descriptions of study procedures, sampling, data collection, and statistical analysis. This improves transparency and reproducibility.

**6. Include sample size justification and power calculations to support statistical power analysis.**

**Response:**
A detailed Sample Size Determination subsection has been added to the Methods section. This includes the assumed prevalence, confidence level, margin of error, statistical power, and final achieved sample size, demonstrating that the study was adequately powered.
